# International survey of antibiotic dosing and monitoring in adult intensive care units

**DOI:** 10.1186/s13054-023-04527-1

**Published:** 2023-06-19

**Authors:** Paul G. Williams, Alexis Tabah, Menino Osbert Cotta, Indy Sandaradura, Salmaan Kanji, Marc H. Scheetz, Sahand Imani, Muhammed Elhadi, Sònia Luque-Pardos, Natalie Schellack, Cristina Sanches, Jean-Francois Timsit, Jiao Xie, Andras Farkas, Kathryn Wilks, Jason A. Roberts, National Coordinators on behalf of the European Society of Intensive Care Medicine (ESICM) and the European Society of Clinical Microbiology and Infectious Diseases (ESCMID) Study Group for Infections in Critically Ill Patients [ESGCIP], Alexander Brinkmann, Mahesh Ramanan, Despoina Koulenti, Mohan Gurjar, Helmi Sulaiman, Gentle Shrestha, Andrea Cortegiani, Monica Crespo, Lowell Ling, Khalid Abidi, Peter Schellongowski

**Affiliations:** 1 Faculty of Medicine, University of Queensland Centre for Clinical Research (UQCCR), The University of Queensland, Brisbane, QLD 4029 Australia; 2 Pharmacy Department, Sunshine Coast University Hospital, Birtinya, QLD Australia; 3 Intensive Care Unit, Redcliffe Hospital, Redcliffe, QLD Australia; 4 Queensland University of Technology, Brisbane, QLD Australia; 5 Sydney Medical School, University of Sydney, Sydney, NSW Australia; 6 Centre for Infectious Diseases and Microbiology, Westmead Hospital, Sydney, NSW Australia; 7 Institute for Clinical Pathology and Medical Research, New South Wales Health Pathology, Sydney, NSW Australia; 8 The Ottawa Hospital and Ottawa Hospital Research Institute, Ottawa, ON Canada; 9 Pharmacometric Center of Excellence, Departments of Pharmacy Practice and Pharmacology, College of Pharmacy, Midwestern University, Downers Grove, IL USA; 10 Nepean Blue Mountains Local Health District, Nepean Hospital, Sydney, NSW Australia; 11 Faculty of Medicine, University of Tripoli, Tripoli, Libya; 12 Pharmacy Department, Parc de Salut Mar, Barcelona, Spain; 13 Infectious Pathology and Antimicrobials Research Group (IPAR), Institut Hospital del Mar d’Investigacions Mèdiques (IMIM), Barcelona, Spain; 14 CIBER of Pharmacy, Saint Clare’s Infectious Diseases (CIBERINFEC CB21/13/0002) Institute of Health Carlos III, Madrid, Spain; 15 Department of Pharmacology, Faculty of Health Sciences, University of Pretoria, Pretoria, South Africa; 16 Campus Centro Oeste Dona Lindu, Federal University of Sao João del Rei, Divinópolis, Minas Gerais Brasil; 17 Assistance Publique Hôpitaux de Paris – Bichat hospital Medical and infectious diseases ICU (MI2), 75018 Paris, France; 18 IAME U 1137 Université Paris-Cité Site Bichat, 75018 Paris, France; 19 Department of Pharmacy, The Second Affiliated Hospital of Xi’an Jiaotong University, Xi’an, China; 20 Optimum Dosing Strategies, Bloomingdale, NJ USA; 21 Department of Pharmacy, Saint Clare’s Health, Denville, NJ USA; 22 Infectious Diseases Department, Sunshine Coast University Hospital, Birtinya, QLD Australia; 23 School of Public Health, The University of Queensland, Brisbane, QLD Australia; 24 Department of Intensive Care Medicine, Royal Brisbane and Women’s Hospital, Brisbane, QLD Australia; 25 Pharmacy Department, Royal Brisbane and Women’s Hospital, Brisbane, QLD Australia; 26 Division of Anaesthesiology Critical Care Emergency and Pain Medicine, Nîmes University Hospital, University of Montpellier, Nîmes, France

**Keywords:** Beta-lactams, Vancomycin, Aminoglycosides, Drug monitoring, Intensive care units

## Abstract

**Background:**

In recent years, numerous dosing studies have been conducted to optimize therapeutic antibiotic exposures in patients with serious infections. These studies have led to the inclusion of dose optimization recommendations in international clinical practice guidelines. The last international survey describing dosing, administration and monitoring of commonly prescribed antibiotics for critically ill patients was published in 2015 (ADMIN-ICU 2015). This study aimed to describe the evolution of practice since this time.

**Methods:**

A cross-sectional international survey distributed through professional societies and networks was used to obtain information on practices used in the dosing, administration and monitoring of vancomycin, piperacillin/tazobactam, meropenem and aminoglycosides.

**Results:**

A total of 538 respondents (71% physicians and 29% pharmacists) from 409 hospitals in 45 countries completed the survey. Vancomycin was mostly administered as an intermittent infusion, and loading doses were used by 74% of respondents with 25 mg/kg and 20 mg/kg the most favoured doses for intermittent and continuous infusions, respectively. Piperacillin/tazobactam and meropenem were most frequently administered as an extended infusion (42% and 51%, respectively). Therapeutic drug monitoring was undertaken by 90%, 82%, 43%, and 39% of respondents for vancomycin, aminoglycosides, piperacillin/tazobactam, and meropenem, respectively, and was more frequently performed in high-income countries. Respondents rarely used dosing software to guide therapy in clinical practice and was most frequently used with vancomycin (11%).

**Conclusions:**

We observed numerous changes in practice since the ADMIN-ICU 2015 survey was conducted. Beta-lactams are more commonly administered as extended infusions, and therapeutic drug monitoring use has increased, which align with emerging evidence.

**Supplementary Information:**

The online version contains supplementary material available at 10.1186/s13054-023-04527-1.

## Background

Treating serious infections in the intensive care unit (ICU) can be complex and challenging [1]. Current antibiotic dosing risks a suboptimal therapeutic response in a large proportion of critically ill patients [2–5]. Additionally, the likelihood of developing antibiotic resistance may increase when specific pharmacokinetic-pharmacodynamic (PK/PD) targets are not achieved [6].

Product information-derived dosing has limited application in critically ill patients given the altered PKs and challenges in pathogen susceptibility to antibiotics, relative to other population groups in the hospital [7]. Dosing modalities available to clinicians to optimize antibiotic dosing in the critically ill are growing, including prolonged infusions for beta-lactams; therapeutic drug monitoring (TDM); and dosing software [8, 9].

International surveys have been conducted to measure the translation of antibiotic optimization research into clinical practice [10–15]. The last large-scale international survey of antibiotic dosing and monitoring in ICUs was published in 2015 (ADMIN-ICU 2015) with respondents describing large variations in clinical practice [10]. Since this time, literature supporting alternative antibiotic dosing strategies has progressed and led to guidelines endorsing PK/PD focused antibiotic dosing [16, 17]. In addition, TDM and dosing software has become more readily available to clinicians [9, 18, 19]. Therefore, an up-to-date survey describing the evolution of practice is highly relevant to understand diversity and uptake of emerging evidence.

The aim of this international cross-sectional study was to survey clinicians working in ICUs worldwide to describe contemporary practices in dosing, administration and monitoring of commonly prescribed antibiotics including glycopeptides, beta-lactams, and aminoglycosides.

## Methods

A panel of international experts formed the writing committee and developed the survey, building upon the foundations of the ADMIN-ICU 2015 survey [10]. The survey included multiple choice and 5-point Likert scale questions, and clinical vignettes to describe contemporary practices used in the dosing, administration and monitoring of commonly prescribed antibiotics for critically ill patients.

The survey was constructed to describe professional characteristics of the respondent. Additionally, the survey collected details of the respondent’s work location and access to resources associated with dosing, administration and monitoring of commonly prescribed antibiotics in the ICU. The country in which respondents worked was categorized by region and economy according to World Bank criteria [20].

The investigators developed clinical vignettes to ascertain information regarding an individual’s practices used for dosing, administering and monitoring of vancomycin, beta-lactams, and the respondent's choice of aminoglycoside. Clinical vignette responses were based on a septic 35-year-old patient, weighing 80 kg with a height of 1.78 m and normal renal function (creatinine clearance = 90 mL/min), with an additional scenario of a 200 kg patient selected for vancomycin. The use of a loading dose (LD), maintenance dose (MD) and frequency, infusion duration, TDM utilization, PK/PD target and dose adjustment method used in clinical practice was surveyed.

A range of infusion durations were defined as presented in Additional file 1: Table S1. The full text of the survey is available in Additional file 1: Table S2.

From August to December 2021, an open invitation to answer the online survey hosted on the Research Electronic Data Capture (REDCap©) platform was accessible to respondents. Invitations to participate were distributed to members of professional societies as listed in the acknowledgements. Additionally, national coordinators for this study distributed the survey via local networks. Distribution strategies were chosen to maximize the breadth of respondents, to provide an adequate international respondent sample. A reminder email was sent after one and three months. A cross-sectional global representation of clinicians involved in the treatment of critically ill infections were the target of this survey. No incentive was offered to respondents to complete the survey.

The data were extracted from REDCap© into Microsoft Excel®. Members from the writing committee (P.W, A.T and J.A.R) conducted a consensus review of all data. Data were excluded from the final analysis as described in Additional file 1: Table S3. The mean value was used when a range rather than a specific value was entered. The exception to this approach was for trough concentrations (the highest value was used), and peak concentrations (the lowest value was used).

Data were expressed as median values with inter-quartile range for continuous variables, and as numbers and/or percentages for categorical variables. Data derived from ADMIN-ICU 2015 were also expressed in this way. A sub-group analysis was performed to determine any difference in TDM utilization or beta-lactam prolonged infusion (continuous or extended infusion) administration between academic hospitals (university or university affiliated) and general hospitals. A Chi-square test was used to make this determination. Descriptive summary statistics were produced (using IBM SPSS Statistics v27) to present survey findings.

## Results

### Demographics

A total of 538 respondents from 409 hospitals in 292 cities in 45 countries completed the survey. Respondents from a variety of regions were represented, including Europe and Central Asia (30%, 163/538), Middle East and North Africa (23%, 126/538), and East Asia and Pacific (17%, 91/538). Most respondents were from a high-income country (HIC) (54%, 288/538), followed by upper-middle-income country (UMIC) (23%, 125/538), with 23% (125/538) from either a low-income country (LIC) or lower-middle-income country (LMIC) (see Additional file 1: Table S4). Table 1 presents respondent demographics, with most being ICU specialists, working in mixed Medical-Surgical closed ICUs within University or University-affiliated Hospitals.

**Table 1:** Respondent demographics

Characteristic	*n* (%)
*Position (n* = *534)*	
**Physicians**	**377 (71)**
Physician in training (ICU)	69 (13)
Physician in training (ID)	31 (6)
Specialist in intensive care medicine	187 (35)
Specialist in infectious diseases	30 (6)
Other	59 (11)
**Pharmacists**	**153 (29)**
ICU pharmacist	96 (18)
ID pharmacist	20 (4)
AMS pharmacist	28 (5)
Other	9 (2)
Others	4 (1)
*Experience in ICU (n* = *533)*	
< 5 years	258 (48)
5–10 years	131 (25)
> 10 years	144 (27)
*Type of hospital (n* = *529)*	
General	188 (36)
Rural	13 (2)
University	199 (38)
University affiliated	123 (23)
Other	6 (1)
*ICU type (n* = *528)*	
Cardiac	9 (2)
Medical	99 (19)
Medical-surgical	342 (65)
Surgical	48 (9)
Other	30 (6)
Open or closed ICU* (n = *532)*	
Closed	339 (64)
Open	193 (37)
*Access to either national or institutional guidelines for antibiotic dosing? (n* = *534)*	
Yes	407 (76)
No	127 (24)
*Access to guidelines for TDM application, interpretation and dose adjustment? (n* = *534)*	
Yes	260 (49)
No	274 (51)

*ICU*, intensive care unit; *ID*, infectious diseases; *TDM*, therapeutic drug monitoring; *n*, number; *%*, percentage

*An open ICU allows any physician to admit patients, while a closed ICU is managed by intensivists

### Clinical vignette results

#### Vancomycin

**Fig. 1:**
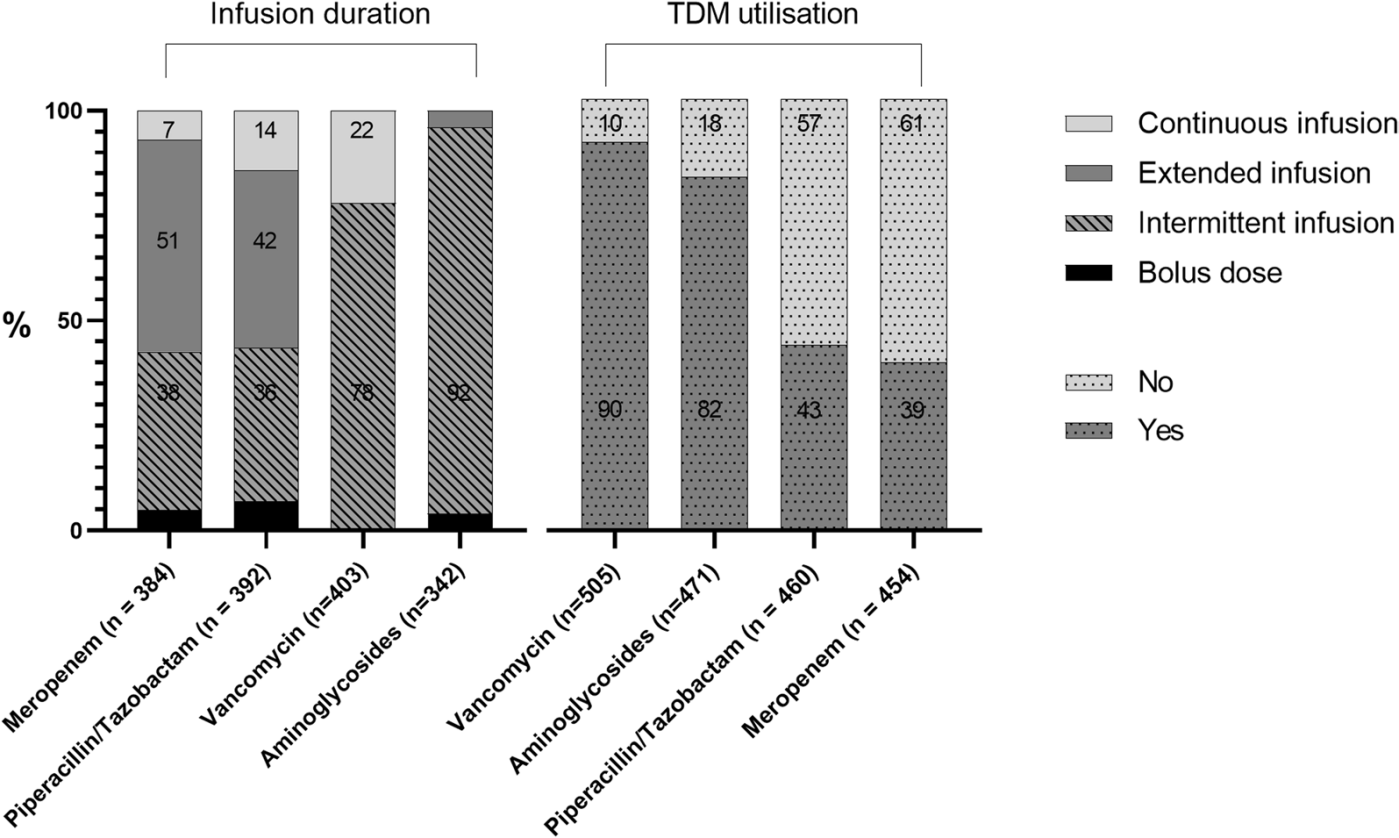
Infusion duration and TDM utilization according to antibiotics. Abbreviations: TDM, therapeutic drug monitoring n, number; %, percentage

**Fig. 2:**
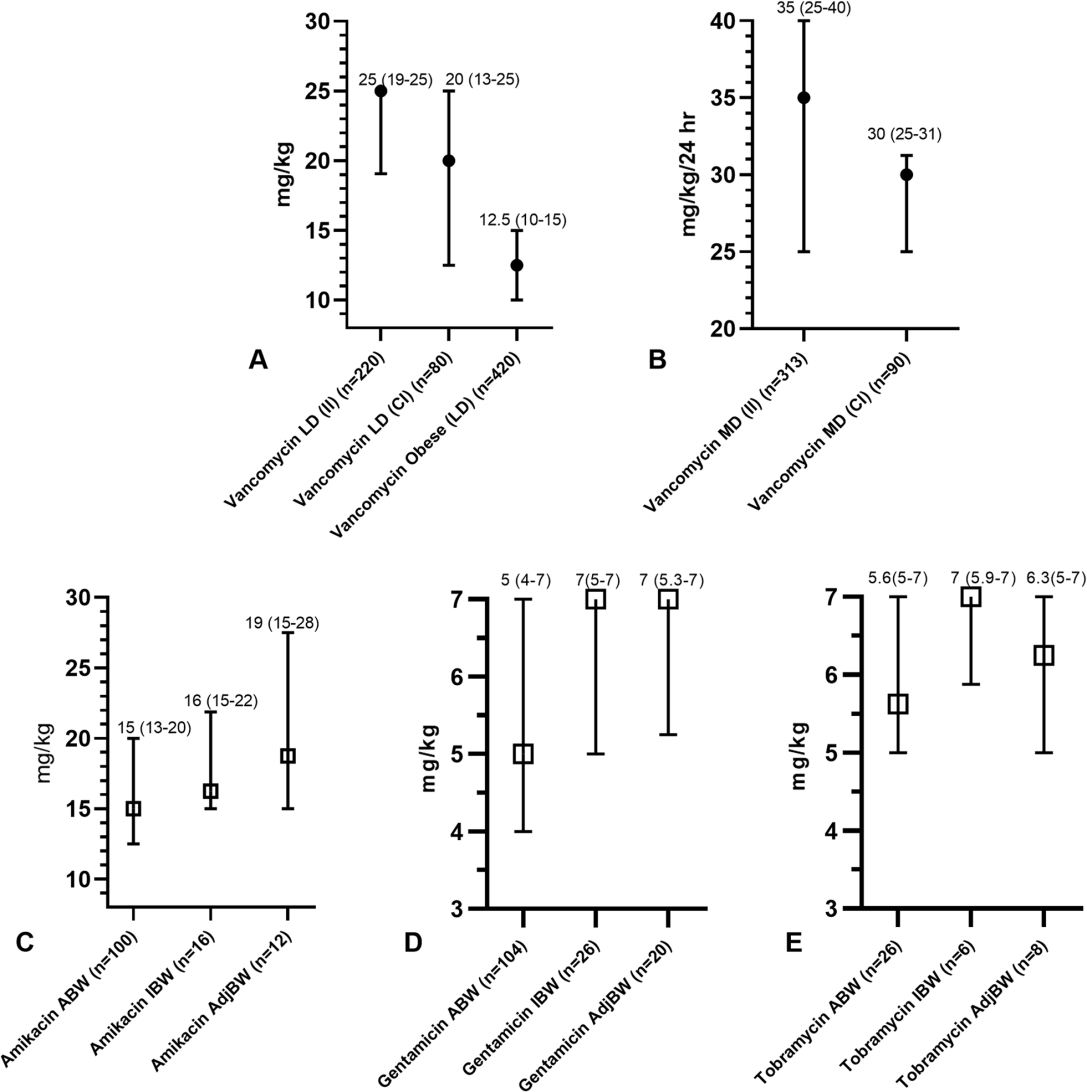
Dosing according to** A**, vancomycin loading dose (LD); **B**, vancomycin maintenance dose (MD); **C**, amikacin*; **D**, gentamicin*; **E**, tobramycin*. Values presented as median (IQR), Abbreviations: IQR, interquartile range; loading dose; II, intermittent infusion, CI, continuous infusion; ABW, actual body weight, IBW, ideal body weight; AdjBW, adjusted body weight; MD; maintenance dose; n, number; *, once daily dosing

Respondents preferenced administering vancomycin as an intermittent infusion (II), with 22% preferencing continuous infusion (CI) (see Fig. 1). Most respondents administered a LD (74%, 300/403), with LDs for II and CI dosing used by 70% (300/403) and 89% (80/90) of respondents, respectively (see Additional file 1: Table S5). The median LD and MD administered for II and CI are presented in Fig. 2. In the 200 kg patient scenario, most respondents capped the LD (72%, 366/508).

Respondents from HICs more frequently administered LDs (80%) as compared to LMICs and LICs (61%, and 17%, respectively). Furthermore, vancomycin II MDs were larger in HICs as compared to LMICs and LICs (see Additional file 1: Table S5). Vancomycin TDM was used by most respondents (90%) (see Fig. 1), and was more often used in HICs (see Additional file 1: Table S5). Respondents from academic hospitals were more likely to perform vancomycin TDM than those from general hospitals (92% vs. 86%, *p* = 0.049), (see Additional file 1: Table S6).

When vancomycin was administered as an II, a trough PK/PD target of 15–20 mg/L was most common, with 21% of respondents targeting an area under the 24 h concentration–time curve (AUC) to minimum inhibitory concentration (MIC) ratio of 400–600 mg·h/L (see Table 2). When vancomycin was administered as an CI, a concentration at steady state (Css) PK/PD target of 20–25 mg/L was most common, with 13% aiming for an AUC/MIC of 400–600 mg·h/L (see Table 2).

**Table 2:** TDM utilization according to antibiotic

Characteristic	Target	*n* (%)
*Vancomycin TDM*		
PK/PD target II (n = 380)	AUC/MIC target 400–600 mg·h/L	80 (21)
	**Trough target**	**300 (79)**
	10–15 mg/L	36 (9)
	12–18 mg/L	31 (8)
	15–20 mg/L	183 (48)
	20–25 mg/L	40 (11)
	Other	10 (3)
PK/PD target CI (n = 69)	AUC/MIC target 400–600 mg·h/L	9 (13)
	**Css target**	**60 (87)**
	20–25 mg/L	45 (65)
	15–20 mg/L	11 (16)
	Other	4 (6)
*Piperacillin/tazobactam TDM*		
PK/PD target (n = 258)	100% *f*T > 4xMIC	59 (23)
	100% *f*T > MIC	92 (36)
	50% *f*T > 4xMIC	46 (18)
	50% *f*T > MIC	33 (13)
	AUC/MIC	11 (4)
	Unsure or other	17 (7)
*Meropenem TDM*		
PK/PD target (n = 204)	100% *f*T > 4xMIC	52 (25)
	100% *f*T > MIC	57 (28)
	50% *f*T > 4xMIC	27 (13)
	50% *f*T > MIC	29 (14)
	AUC/MIC	9 (4)
	Unsure or other	18 (9)
*Aminoglycoside TDM*		
PK-PD target (n = 385)	100% *f*T > MIC	24 (6)
	AUC/MIC	65 (17)
	CMax/MIC	166 (43)
	Unsure	130 (34)
1^st^ TDM sample (n = 387)	Peak	144 (37)
	Trough	205 (53)
	Other	41 (11)
Peak and trough sample taken? (n = 144)	Yes	108 (75)
	No	36 (25)

*TDM*, therapeutic drug monitoring; *PK*, pharmacokinetics; *PD*, pharmacodynamics; *II*, intermittent infusion; *AUC*, area under the curve; *MIC*, minimum inhibitory concentration; *CI*, continuous infusion; *EI*, extended infusion; *Css*, concentration at steady state; *fT*, free time; *CMax*, concentration maximum; *n*, number; *%*, percentage

Vancomycin dose adjustments were most frequently determined by the clinical judgement of the treating physician (57%), by dosing guidelines (33%) and by linear adjustment post-TDM (31%) (see Table 3).

**Table 3:** Dose adjustment method according to antibiotics

Survey question	Response	*n* (%)
Vancomycin doses are generally adjusted by: *(select all that apply)* (n = 535)	Clinical judgement of treating physician	307 (57)
	Linear adjustment following TDM	167 (31)
	Dosing software	60 (11)
	Dosing guideline	178 (33)
	Other	25 (5)
Beta-lactam doses are generally adjusted by: *(select all that apply)* (n = 506)	Clinical judgement of treating physician	290 (57)
	Product information	159 (31)
	TDM	74 (15)
	Dosing software	15 (3)
	Dosing guideline	129 (25)
	Pharmacist recommendation	156 (31)
	ID/AMS recommendation	123 (24)
	Other	24 (5)
Aminoglycoside doses are generally adjusted by: *(select all that apply)* (n = 497)	Clinical judgement of treating physician	256 (52)
	Product information	102 (21)
	TDM	178 (36)
	Dosing software	38 (8)
	Dosing guideline	91 (18)
	Pharmacist recommendation	194 (39)
	ID/AMS recommendation	107 (22)
If the peak concentration is below your target, do you: *(select all that apply)* (n = 325)	Increase the next daily dose	179 (55)
	Leave the daily dose unchanged	19 (6)
	Administer a supplementary dose	38 (12)
	Use dosing software to guide therapy	45 (14)
	Unsure	62 (19)
If the trough concentration is above your target, do you: *(select all that apply)* (n = 362)	Leave the daily dose unchanged	44 (12)
	Decrease the next daily dose	137 (38)
	Delay next dose until the trough is below the target concentration	78 (22)
	Extend the dosing frequency	120 (33)
	Use dosing software to guide therapy	43 (12)

*TDM*, therapeutic drug monitoring; *ID*, infectious diseases; *AMS*, antimicrobial stewardship; *n*, number; *%*, percentage

#### Piperacillin/tazobactam

Piperacillin/tazobactam was most often administered as an extended infusion (EI) (42%) followed by II (36%) and CI (14%) (see Fig. 1). The use of prolonged infusions was comparable between academic and general hospitals (see Additional file 1: Table S6). A LD was preferred by respondents administering an EI (62%) or CI (84%). Regardless of infusion duration, the median LD and MD per 24 h was 4.5 g and 18.5 g, respectively. TDM was performed by 43% of respondents (see Fig. 1) with most measuring a trough concentration at steady state. TDM was performed most often in Sub-Saharan Africa (71%), and Europe and Central Asia (60%), with only 7% usage in North America (see Additional file 1: Table S5). Respondents from academic hospitals were more likely to perform TDM than those from general hospitals (50% vs. 34%, *p* = 0.001), (see Additional file 1: Table S6). The most common PK/PD target was 100% time where the unbound concentration remained above the MIC as a percentage of the dosing interval (100% *f*T > MIC) (36%), followed by 100% *f*T > 4xMIC (23%). When assessing EI and CI only, the most common PK/PD target remained 100% *f*T > MIC.

#### Meropenem

Most respondents preferred to administer meropenem as an EI (51%), followed by II (38%) and CI (7%) (see Fig. 1). The use of prolonged infusions was comparable between academic and general hospitals (see Additional file 1: Table S6). Most respondents preferred a loading dose for EI and CI (62% and 92%, respectively) commonly dosed at 2 g. The median MD per 24 h according to infusion duration is presented in Additional file 1: Table S7. TDM was performed by 39% of respondents (see Fig. 1), with most measuring a trough concentration at steady state. TDM was performed most often in Europe and Central Asia (58%) and South Asia (55%), with only 7% usage in North America (see Additional file 1: Table S5). Respondents from academic hospitals were more likely to perform TDM than those from general hospitals (44% vs. 32%, *p* = 0.014), (see Additional file 1: Table S6). The most common PK/PD target was 100% *f*T > MIC (28%,) followed by 100% *f*T > 4xMIC (25%) (see Table 2). When assessing EI and CI only, the most common PK/PD target remained 100% *f*T > MIC.

Beta-lactam dose adjustments were most frequently determined by the clinical judgement of the treating physician (57%), followed by product information (31%) and pharmacist recommendation (31%) (see Table 3).

#### Aminoglycosides

Most respondents preferred to administer aminoglycosides as a once daily infusion (85%, 331/390) over 30–60 min (II) (92%, 314/342). Gentamicin was the preferred aminoglycoside prescribed (47%, 149/317), followed by amikacin (40%, 128/317), and tobramycin (13%, 40/317). Once daily dosing of aminoglycosides was based on actual body weight by most respondents in our 80 kg patient scenario, with dosing strategies presented in Fig. 2.

TDM was performed by 82% of respondents (see Fig. 1). TDM was most frequently performed in HICs (90%) (see Additional file 1: Table S5); however, utilization was comparable between academic and general hospitals (see Additional file 1: Table S6). Most respondents (53%) recommended the first sample be taken as a trough, and 37% recommended a peak. When a peak was recommended, 75% of respondents would sample a peak and trough (see Table 2).

The median peak and trough concentration targets are presented in Additional file 1: Table S7. If the peak concentration was below the target peak, 45% of respondents would not increase the subsequent dose. Conversely, if the trough concentration was above the target trough, most respondents would either decrease the subsequent dose (38%), or extend the dosing frequency (33%) (see Table 3). The most common PK/PD target was peak-to-MIC ratio (Cmax/MIC) (43%), and 34% of respondents were unsure of the PK/PD target (see Table 2). Clinical judgement of the treating physician was the most common method of adjusting aminoglycoside doses (52%), followed by Pharmacist recommendation (39%) (see Table 3).

#### Dosing software

Dosing software was used by 11%, 3%, and 8% of respondents to adjust vancomycin, beta-lactam, and aminoglycoside doses, respectively (see Table 3). For vancomycin, dosing software was more commonly used in North America (21%), and in HICs (17%). For beta-lactams, dosing software was most frequently used in Europe and Central Asia (5%), and in LMICs (5%). Dosing software utilization for aminoglycoside therapy was most prevalent in East Asia and the Pacific (18%), and in HICs (12%) (see Additional file 1: Table S5). When dosing software was used for vancomycin, 48% of respondents targeted an AUC/MIC ratio, while the majority (52%) preferenced a trough target. A Cmax/MIC target was preferred by respondents using dosing software to guide aminoglycoside therapy (56%), as opposed to AUC/MIC target (44%).

## Discussion

We describe the effectiveness of knowledge translational antibiotic dosing and monitoring strategies used to treat serious infections as the evidence has accumulated over the last 7-years. We found increased use of vancomycin and beta-lactam LDs, and that vancomycin trough or Css PK/PD targets are preferred. HICs are more likely to administer a vancomycin LD and perform TDM for vancomycin and aminoglycoside therapy. Beta-lactam infusions are now predominantly administered as an EI. TDM is increasing in clinical practice, especially in academic hospitals. Dosing software is uncommonly used.

### Vancomycin

Vancomycin LDs are recommended to achieve rapid target attainment and are supported in the 2020 Consensus Guideline of vancomycin therapeutic monitoring for serious MRSA infections [21]. It is concerning that 25% of respondents omitted a LD, and that LD omissions were even more apparent in respondents from LMICs and LICs. The LD specified by respondents did conform with the 2020 guideline recommendation; however, in our obese patient scenario, the median LD was less than the capped loading dose recommended in the Consensus Guideline (3000 mg) and may delay achieving adequate vancomycin exposure [21]. In comparison with ADMIN-ICU 2015, we found an increased use of vancomycin LDs (74% vs 65%) (see Additional file 1: Table S7) [10]. Our findings suggest that strategies to increase the use of appropriate vancomycin LDs are required.

Although respondents from HICs administered larger MDs, all median MDs aligned with the consensus guideline dosage range, regardless of the economy of the country [21].

Most respondents administered vancomycin as an II, which aligns with contemporary guidelines [21]. In comparison with ADMIN-ICU 2015, the most common method of administration remains II; however, CI usage has decreased from 31 to 22% (see Additional file 1: Table S7). Our findings on vancomycin infusion duration were similar to a recent survey of German ICUs [22]. However, other recent surveys have reported prolonged or continuous vancomycin infusion use in approximately 50% of cases [23, 24].

TDM was the preferred dosing strategy (90%) to guide vancomycin dosing in our study. This aligns with the Consensus Guideline [21], and is an increase from 82% in ADMIN-ICU 2015 (see Additional file 1: Table S7) [10]. Our findings were similar to a 2020 survey, which found that 89% of respondents used TDM to guide vancomycin dosing [23]. The majority of respondents in the 2020 survey were from teaching hospitals, and we found increased use of TDM in respondents from academic hospitals. Other surveys have shown rates of TDM use closer to 75%, where the proportion of respondents from teaching hospitals are either lower or unknown [22, 25].

Interestingly, only 11% of respondents used dosing software, even though it is the recommended approach in the Consensus Guideline and has been shown to improve target attainment and reduce nephrotoxicity compared to a trough-guided approach [21, 26]. Adoption of a dosing software approach was most prevalent in North America (21%), which may reflect early uptake of the Consensus Guideline; which was developed by American professional societies [21].

The vancomycin PK/PD target for *S. aureus* infections is established as an AUC/MIC of 400–600 mg·h/L [27]. However, historically, a trough target has been supported by guidelines [21, 27]. This recommended shift in approach was not routinely observed in our study, with most respondents targeting either a trough or Css range. This finding is not unexpected given the challenges clinicians likely face to implement AUC/MIC monitoring in clinical practice [28]. As with dosing software uptake, an AUC/MIC approach was most frequently used in North America (34%), again likely due to the American-derived Consensus Guidelines [21].

### Beta-lactams

Beta-lactam LDs were mostly used for EI and CIs, which aligns with the 2021 surviving sepsis campaign recommendation [16]. However, nearly 40% of respondents did not recommend a LD prior to administering an EI, which may delay adequate drug exposure [29]. We found a significant shift toward beta-lactam EI use, which aligns with contemporary evidence, guideline recommendations and is associated with a short-term mortality benefit [16, 18, 30, 31]. Prolonged infusions were consistently favoured in both academic and general hospitals. Other recent surveys of mostly European or Columbian respondents also favoured prolonged infusion of beta-lactams [23, 25]. However, findings from national surveys in England and China favoured intermittent infusions [24, 32]. Variability in infusion duration was seen in our study depending on the region, highlighting inconsistency in practice globally. In comparison with ADMIN-ICU 2015 findings, piperacillin/tazobactam EI and CI use has doubled, and meropenem EI use has increased from 28 to 51% (see Additional file 1: Table S7).

Uptake of beta-lactam TDM varied markedly depending on region, with large variability observed even within HICs. However, TDM utilization was more prevalent in academic hospitals. Future exploration of contributing factors to such variability should be undertaken. The use of TDM has increased from approximately 10% in ADMIN-ICU 2015 to around 40% in our study (see Additional file 1: Table S7) [10]. Recent largely European surveys reported 17–52% of respondents had access to beta-lactam TDM [22, 23, 33], with one French survey reporting systematic beta-lactam TDM use in 38% of respondents [33]. Increased uptake of beta-lactam TDM likely reflects the emerging evidence of a TDM-guided approach in terms of achieving pre-defined therapeutic concentrations and its inclusion in contemporary guidelines to treat serious infections [16, 18]. However, definitive clinical evidence supporting a TDM approach is still lacking in terms of patient outcomes [34].

Only 3% of respondents used dosing software to guide beta-lactam therapy. Besides a recent survey that reported 26.8% beta-lactam dosing software use in Columbian ICUs [25], uptake of beta-lactam dosing software use remains to be determined. The evidence to support a dosing software strategy is sparse; however, promising results in terms of achieving PK/PD targets have been demonstrated [9], and further investigation is required.

The most common PK/PD target for beta-lactams (regardless of infusion duration) was 100% *f*T > MIC. This target has been associated with improved clinical efficacy compared to lower drug exposure targets in patients with serious infections and is the supported PK/PD target in a recent international guideline for the management of sepsis and septic shock [4, 16].

### Aminoglycosides

Aminoglycosides were mostly administered as a once daily II, which aligns with contemporary guidelines [16, 18, 35]. However, 15% of respondents preferred divided daily dose administration, and risks ototoxicity and nephrotoxicity [36]. In comparison with our results, growing evidence supports even higher aminoglycoside doses in patients with serious infections to achieve adequate peak concentrations [37–40].

TDM-guided aminoglycoside dosing is recommended in contemporary guidelines [16, 18]. In our study, 82% of respondents used TDM to guide aminoglycoside dosing, with similar rates observed in both academic and general hospitals. The use of TDM for aminoglycosides remains similar to the findings of the ADMIN-ICU 2015 survey (see Additional file 1: Table S7) and is consistent with the findings of a recent survey [23]. Dosing software, however, was only used by approximately 8% of respondents, with highest usage in HICs and in the East Asia and Pacific region (18%). Contemporary guidelines recommend the use of dosing software [18], and this dosing strategy has been shown to reduce both hospital length of stay and rates of nephrotoxicity [41]. Further evaluation of this dosing strategy is required.

Our results showed that a Cmax/MIC target for aminoglycosides was most frequently used, with aminoglycoside peak and trough targets comparable with the ADMIN-ICU 2015 findings (see Additional file 1: Table S7) [10]. However, the peak targets were insufficient to achieve a Cmax/MIC ratio of > 8–10, which is a well-established marker of clinical success for aminoglycoside dosing [42, 43]. Of note, we observed that 34% of respondents were unsure of the PK/PD targets for aminoglycosides which may be due to decreasing use of aminoglycosides in favour of other agents over recent years.

There are several limitations to our study. Firstly, the electronic survey was broadly distributed, with no means to measure how many health professionals had the opportunity to participate in the survey, and no means to measure the response rate. Therefore, non-response bias may be present and skew the results. However, the large sample across many institutions and countries may minimize this bias.

Secondly, generalizing the results is difficult given the global regions' weighting. To mitigate this limitation, the authors ensured the survey was distributed widely via professional bodies and national coordinators, through the various regions.

Thirdly, de-duplication of respondents from the same hospital has not been possible and may influence the results presented. However, the focus of this study was primarily on the individual clinician’s practice and given the number of hospitals represented as a percentage of total respondents (409/538, 76%), this influence is unlikely meaningful.

Fourthly, it is plausible, although unlikely, that changes in practice observed in this study as compared to ADMIN-ICU 2015 were due to sampling a different cohort of respondents, as opposed to representing practice change over time. All data collected were de-identified as required during our ethics approval process. Therefore, it was not possible to report respondents who completed both ADMIN-ICU surveys. However, the survey samples are large enough to infer that they are likely representative of true practice.

Fifthly, the use of a simple clinical vignette may have limited the generalizability of our findings. For example, if a patient exhibited augmented renal clearance or developed renal failure, respondents may have been more likely to recommend TDM or prolonged infusions. The simple clinical vignette was selected to be consistent with ADMIN-ICU 2015 in order to compare findings.

Finally, the survey responses may not align with those made in clinical practice based on an actual patient and their inevitable complexities. However, a key objective of this study was to characterize changes in practice since the ADMIN-ICU 2015, which was subject to the same limitations and hence comparable. Although, differences in respondent demographics were apparent in the present study, with less experienced respondents and more pharmacists included.

## Conclusions

Despite these limitations, importantly, we present contemporary dosing, administration and monitoring of a selection of antibiotics used for serious infections and highlighted changes in practice over the last 7-years and how current practice aligns with contemporary evidence and guidelines. Although large variation in dosing and monitoring practices were observed, there have been significant clinical practice changes aligning with contemporary evidence and guidelines. Most notably, beta-lactams are now most commonly administered as an EI, with TDM use increasing in clinical practice. The authors believe that observational methods could be used to better understand current antibiotic dosing practices in this patient cohort on a global scale. This would increase confidence in the findings of this study. Additionally, future research is needed to assess the impact of antibiotic dosing strategies on important patient-centred outcomes.

## Supplementary Information

**Additional file 1.** Supplementary Material, Description of data: **Table S1.** Definition of infusion types and duration; **Table S2.** Online survey questions; **Table S3.** Exclusions; **Table S4.** Respondents as per Region and Economy; **Table S5.** Results according to Region and Economy; **Table S6.** TDM utilization and beta-lactam prolonged infusion administration according to hospital type.

## Data Availability

All data generated or analyzed during this study are included in this published article [and its supplementary information files].

## Abbreviations

ICU: Intensive care unit
PK: Pharmacokinetics
PD: Pharmacodynamics
TDM: Therapeutic drug monitoring
LD: Loading dose
MD: Maintenance dose
REDCap©: Research electronic data capture
HIC: High-income country
UMIC: Upper-middle-income country
LMIC: Lower-middle-income country
LIC: Low-income country
II: Intermittent infusion
CI: Continuous infusion
AUC: Area under the 24-h concentration–time curve
MIC: Minimum inhibitory concentration
EI: Extended infusion
%*f*T > MIC: % Time where the unbound concentration remained above the MIC as a percentage of the dosing interval
Cmax: Peak concentration
Css: Concentration at steady state
