# Supplementary material for: International survey of antibiotic dosing and monitoring in adult intensive care units
Source: Crit Care. 2023 Jun 19;27:241. doi: 10.1186/s13054-023-04527-1 (PMC10278304; doi:10.1186/s13054-023-04527-1)
Supplement: Supplementary file 1 — Additional file 1. Supplementary Material, Description of data: Table S1. Definition of infusion types and duration; Table S2. Online survey questions; Table S3. Exclusions; Table S4. Respondents as per Region and Economy; Table S5. Results according to Region and Economy; Table S6. TDM utilization and beta-lactam prolonged infusion administration according to hospital type. [file 13054_2023_4527_MOESM1_ESM.pdf]

Supplementary material

Contents

Supplementary material 1
Table S1: Definition of infusion types and duration 2
Table S2: Online survey questions 3
Table S3: Exclusions 12
Table S4: Respondents as per Region and Economy 13
Table S5: Results according to Region and Economy 14
Table S6: TDM utilization and beta-lactam prolonged infusion administration according to hospital type 21
Table S7: Comparable results (ADMIN ICU 2021 and ADMIN ICU 2015) 22

Table S1: Definition of infusion types and duration

Antibiotic	Infusion type	Infusion duration
Vancomycin	Intermittent infusion	< 24 hours
	Continuous infusion	24 hours
Beta-lactams	Bolus	< 10 mins
	Intermittent infusion	10 – 120 mins
	Extended infusion	> 2 hours
	Continuous infusion	24 hours
Aminoglycosides	Bolus	< 10 mins
	Intermittent infusion	30 – 60 mins
	Extended infusion	> 60 mins

Table S2: Online survey questions

Part 1: Demographics and General Information

Country:
City/town:
Hospital name:

Type of hospital

- University
- University affiliated
- General
- Rural

Type of ICU

- Medical
- Surgical
- Medical-Surgical
- Cardiac
- Other (please specify)

Open or closed

- Open
- Closed

Your health profession

- Pharmacist
- Doctor
- Nurse
- Other (please specify)

Current position

- Specialist in Intensive Care medicine
- Specialist in Infectious Diseases
- Intensive Care Unit (ICU) Pharmacist
- Infectious Diseases (ID) Pharmacist
- Antimicrobial Stewardship (AMS) Pharmacist
- ICU Nurse
- AMS Nurse
- Doctor in training – ICU
- Doctor in training – ID
- Other (please specify)

How many years' experience do you have in your current position?

- < 5 years
- 5-10 years
- 11-20 years
- > 20 years

Level of ID input in the ICU

- None
- At least one ICU practitioner is qualified in Clinical Microbiology or ID
- Consultation on request only
- Regular ID round or consultation

If regular ID/Micro round consultation, how many consultations/rounds per week?

- 1
- 2
- 3
- 4
- 5
- 6
- 7
- Not applicable

Members of the infectious disease team rounding/consulting (Select all that apply)

- ID Specialist
- Doctor in training – ID
- ID/AMS Pharmacist
- ID/AMS Nurse
- Microbiologist
- Other (please specify)

Availability of an ICU pharmacist

- None
- Phone consultation
- Available in the ICU 7-days per week
- Available in the ICU Monday to Friday during business hours.
- Other (please specify)

Do you have national guidelines for antibiotic drug dosing?

- No
- Yes but not strictly followed
- Yes and strictly followed

Do you have institutional guidelines for antibiotic drug dosing?

- No
- Yes but not strictly followed
- Yes and strictly followed

Do you have TDM guidelines for TDM application, interpretation and dose adjustment?

- No
- Yes but not strictly followed
- Yes and strictly followed

Part 2: Clinical Vignettes

The following clinical vignettes are hypothetical and the intention is for respondents to answer even if they do not have access to/perform TDM

Glycopeptides

Which glycopeptide is most commonly used in your unit?

- Vancomycin
- Teicoplanin

Please answer the remaining glycopeptide questions for vancomycin

Vancomycin is most commonly used in your ICU as:

- A short infusion regardless of dose (infusion less than 2 hours)
- An extended infusion (maximum rate of 600 mg per hour)
- An extended infusion (maximum rate of 1000 mg per hour)
- A continuous infusion

In general in your ICU, vancomycin TDM is used for: (Select all that apply)

- All patients (regardless of duration of therapy)
- All patients on therapy for ≥ 48 hours
- Patients with renal failure
- Patients with suspected toxicity
- Patients with suspected augmented renal clearance
- Infrequently
- Never
- Unsure
- Other (please specify)

In your ICU, how are vancomycin doses adjusted? (Select all that apply)

- Clinical judgement
- Linear adjustment following TDM (i.e. double dose if level is half what it should be)
- TDM + dose optimisation software (please specify software)
- As per endorsed dosing guideline
- Other (please specify)

Please enter all values as you would recommend for a septic 35-year-old patient, weighing 80kg with a height of 1.78m with a normal renal function (CrCl = 90mL/min)

In this scenario would you recommend a loading dose of vancomycin?

- Yes
- No
- Unsure

If yes, what dose and duration of infusion would recommend?

- dose in mg
- or dose in mg/kg (actual body weight)
- or dose in mg/kg (ideal body weight)
- duration of the infusion (in hours)

What maintenance dose would you recommend?

- Dose in mg/kg
- Or dose in mg
- Dose frequency in hours (i.e. 12)
- Duration of the infusion (in hours)

In this scenario, the first vancomycin TDM blood sample would be taken:

- After the loading dose
- As a trough before the 3rd dose
- As a trough before the 4th dose
- As a random concentration around 24 hours post commencement of a continuous infusion
- I would not recommend TDM (please select 'Not applicable' for the following question)
- Other (please specify)

What pharmacodynamic target would you aim for?

- A calculated area under the curve (AUC)/MIC of 400
- Trough concentration range of 12-18 mg/L
- Trough concentration range of 15-20 mg/L
- A concentration range of 20-25 mg/L 24-hours post commencement of a continuous infusion
- Trough concentration range of 10-15 mg/L
- Other (please specify)
- Not applicable

If the patient weighed 200kg would your loading dose be capped?

- Yes
- No

What would your loading dose be in this 200kg patient?

- dose in mg
- or dose in mg/kg (actual body weight)
- or dose in mg/kg (ideal body weight)
- duration of the infusion (in hours)
- other (please specify)

Beta-lactams (e.g. Penicillins, Cephalosporins, Carbapenems)

In your ICU, Beta-lactam TDM is used for: (Select all that apply)

- All patients
- Patients with renal failure
- Patients with suspected augmented renal clearance
- Patients with suspected toxicity
- Infrequently
- Never
- Unsure
- Other (please specify)

In your ICU, how are Beta-lactam doses primarily adjusted? (Select all that apply)

- Clinical judgement of treating doctor
- As per product information recommendations (i.e. dosing in renal impairment)
- TDM
- TDM + dose optimisation software (please specify program used)
- As per endorsed dosing guideline
- Pharmacist recommendation
- ID/AMS recommendation
- Other (please specify)

Piperacillin / Tazobactam

Piperacillin/Tazobactam is most commonly administered in your ICU as:

- A bolus dose (less than 10 minutes)
- A short infusion (infusion less than 2 hours)
- An extended infusion (infusion between 2 – 4 hours)
- A continuous infusion

Please enter all values as you would recommend for a septic 35-year-old patient, weighing 80kg with a height of 1.78m with a normal renal function (CrCl = 90mL/min)

In this scenario would you recommend a loading dose of piperacillin/tazobactam?

- Yes
- No
- Unsure

If yes, what dose would you recommend? (if no, please skip this question)

- dose in grams (state combined dose e.g. 4.5 g)
- or dose in mg/kg
- duration of the infusion (in minutes)

What maintenance dose would you recommend?

- Dose in grams (state combined dose e.g. 4.5 g)
- Dose frequency in hours
- Duration of the infusion (in minutes)

In this scenario, the first TDM blood sample would be taken:

- After the loading dose
- As a trough approximately 24 hours after commencing therapy
- As a trough approximately 48 hours after commencing therapy
- As a random concentration approximately 24 hours post commencement of a continuous infusion
- I would not recommend TDM (please select 'Not applicable' for the next 2 questions)
- Other (please specify)

What pharmacodynamic target would you aim for?

- 100% *f*T>MIC (100% time above the MIC)
- 50% *f*T>MIC (50% time above the MIC)
- 50% *f*T>4xMIC (50% time above four times the MIC)
- 100% *f*T>4xMIC (100% time above four times the MIC)
- CMax/MIC
- AUC/MIC
- Other (please specify)
- Not applicable

What trough concentration would you aim for?

- ____ mg/L
- Not applicable

Meropenem

Meropenem is most commonly administered in your ICU as:

- A bolus dose (less than 10 minutes)
- A short infusion (infusion less than 2 hours)
- An extended infusion (infusion between 2 – 4 hours)
- A continuous infusion

Please enter all values as you would recommend for a septic 35-year-old patient, weighing 80kg with a height of 1.78m with a normal renal function (CrCl = 90mL/min)

In this scenario would you recommend a loading dose of meropenem?

- Yes
- No
- Unsure

If yes, what dose would you recommend? (if no, please skip this question)

- dose in grams
- or dose in mg/kg
- duration of the infusion (in minutes)

What maintenance dose would you recommend?

- dose in grams
- Dose frequency in hours
- Duration of the infusion (in minutes)

In this scenario, the first TDM blood sample would be taken:

- After the loading dose
- As a trough around 24 hours after commencing therapy
- As a trough around 48 hours after commencing therapy

- As a random concentration around 24 hours post commencement of a continuous infusion
- I would not recommend TDM (please select 'Not applicable' for the next 2 questions)
- Other (please specify)

What pharmacodynamic target would you aim for?

- 100% *f*T>MIC (100% time above the MIC)
- 50% *f*T>MIC (50% time above the MIC)
- 50% *f*T>4xMIC (50% time above four times the MIC)
- 100% *f*T>4xMIC (100% time above four times the MIC)
- CMax/MIC
- AUC/MIC
- Other (please specify)
- Not applicable

What trough concentration would you aim for?

- ____ mg/L
- Not applicable

Aminoglycosides

Which aminoglycoside is the most commonly used in your ICU?

- Amikacin
- Gentamicin
- Tobramycin
- Other

This aminoglycoside is most commonly administered in your ICU as:

- A bolus dose (less than 10 minutes)
- A short infusion (30 – 60 mins)
- Other (please specify)

This aminoglycoside is most commonly prescribed in your ICU as a:

- Once daily dose
- Multiple daily dose (specify number of doses per day 2-4)

What is the target peak concentration for the most commonly used aminoglycoside in your practice?

- _____mg/L
- Not applicable

If the peak concentration is below that number, do you

- Increase the daily dose for the next infusion
- Leave the daily dose unchanged
- Re-administer a supplementary dose as soon as you receive the result
- Use dose optimisation software to guide therapy
- Unsure
- Not applicable

What is your target trough concentration for the most commonly used aminoglycoside in your practice?

- _____mg/L
- Not applicable

If the trough concentration is above that number, do you

- Leave the daily dose unchanged
- Decrease the daily dose for the next infusion
- Sample trough levels later and don't readminister until below that number
- Extend the dosing frequency
- Use dose optimisation software to guide therapy
- Not applicable

In your ICU, how are Aminoglycoside doses generally adjusted? (Select all that apply)

- Clinical judgement of treating doctor
- As per product information recommendations (i.e. dosing in renal impairment)
- TDM
- TDM + dose optimisation software (please specify program used)
- As per endorsed dosing guideline
- Pharmacist recommendation
- ID/AMS recommendation
- Other (please specify)

Please enter all values as you would recommend for a septic 35-year-old patient, weighing 80kg with a height of 1.78m with a normal renal function (CrCl = 90mL/min)

In this scenario, what dose of the most commonly prescribed aminoglycoside would you recommend?

- dose in mg
- or dose in mg/kg (actual body weight)
- or dose in mg/kg (ideal body weight)
- or dose in mg/kg (adjusted body weight)
- duration of the infusion (in minutes)

In this scenario, the first Aminoglycoside TDM blood sample would be taken:

- As a peak concentration at the completion of the first dose
- As a trough around 24 hours after commencing therapy
- As a trough around 48 hours after commencing therapy
- As a peak concentration after the completion of the 3rd dose
- As a random sample after the 3rd dose
- I would not recommend TDM (please select 'Not applicable' for the next question)
- Other (please specify)

In this scenario, a second blood sample would be taken during the dosing interval and used for dose monitoring (i.e. to measure or predict trough and/or AUC)?

- Yes
- No

What pharmacodynamic target would you aim for?

- CMax/MIC
- AUC/MIC
- 100% *f*T>MIC (100% time above the MIC)
- Unsure
- Not applicable

Do you certify this data to be a complete and accurate and would you like your response to be recorded?

- Yes, my response is complete and accurate and should be recorded
- No, I was just testing the survey, do not record my response
- No, do not record this response (other reasons) – please specify

Table S3: Exclusions

Exclusion criteria	Exclusion frequency (%)	Total respondents included
Respondents did not certify their response as complete	190/912, (21%)	
Respondent did not hold the qualifications necessary to practice (e.g. unlicensed student)	89/722, (12%)	
≥ 50% of the respondent's answers were missing	84/633, (13%)	
≥ 50% of the respondent's numerical value answers were well outside the expected parameters (e.g. gentamicin dosed 30mg every 8 hours)*	7/549, (1.3%)	
Duplicate entries existed (only the last entry was included)	4/542, (0.7%)	538

* *When less than half of the respondent's numeric responses were well outside the expected parameters, those responses were excluded from the analysis. The remaining data was included in the analysis*

Table S4: Respondents as per Region and Economy

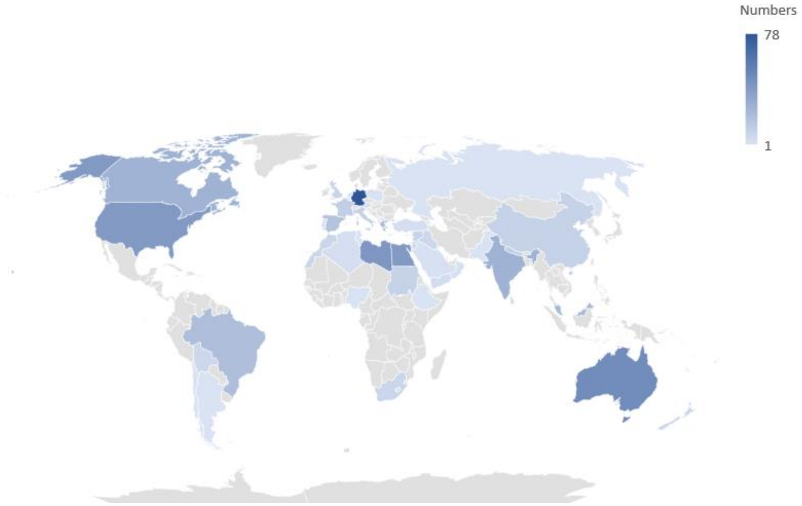

Country by Region	n (%)	Country by Region	n (%)
East Asia and Pacific	91 (17%)	Middle East and North Africa	126 (23%)
Australia	48	Iraq	7
New Zealand	7	Jordan	7
China	10	Lebanon	1
Taiwan	3	Oman	2
Malaysia	23	Saudi Arabia	2
Europe and Central Asia	163 (30%)	Syria	9
Albania	1	Yemen	4
Greece	23	Algeria	3
Italy	11	Egypt	40
Portugal	1	Libya	42
Spain	18	Morocco	8
Poland	1	Tunisia	1
Russia	1	North America	67 (12%)
Austria	2	Canada	27
Belgium	1	United States	40
France	12	South Asia	41 (8%)
Germany	78	India	27
Netherlands	2	Nepal	13
United Kingdom	9	Pakistan	1
Turkey	3	Sub-Saharan Africa	20 (4%)
Latin America and the Caribbean	30 (6%)	Ethiopia	1
Argentina	1	Nigeria	1
Bolivia	7	Sudan	10
Brazil	21	South Africa	8
Chile	1	Total	538
**Country by Economy**	**n (%)**	**Country by Economy**	**n (%)**
Low-Income	24 (4%)	Russia	1
Ethiopia	1	Malaysia	23
Sudan	10	South Africa	8
Syria	9	Turkey	3
Yemen	4	High-Income	288 (54%)
Lower-Middle-Income	101 (19%)	Australia	48
Algeria	3	Austria	2
Bolivia	7	Belgium	1
Egypt	40	Canada	27
India	27	Chile	1
Morocco	8	France	12
Nepal	13	Germany	78
Nigeria	1	Greece	23
Pakistan	1	Italy	11
Tunisia	1	Netherlands	2
Upper-Middle-Income	125 (23%)	New Zealand	7
Albania	1	Oman	2
Argentina	1	Poland	1
Brazil	21	Portugal	1
China	10	Saudi Arabia	2
Iraq	7	Spain	18
Jordan	7	Taiwan	3
Lebanon	1	United Kingdom	9
Libya	42	United States	40
Total			538

Table S5: Results according to Region and Economy

Variable	Total	East Asia and Pacific	Europe and Central Asia	Latin America and the Caribbean	Middle East and North Africa	North America	South Asia	Sub-Saharan Africa	High-Income	Upper-Middle - Income	Lower-Middle-Income	Low-Income
	n (%)	n (%)	n (%)	n (%)	n (%)	n (%)	n (%)	n (%)	n (%)	n (%)	n (%)	n (%)
**Position**	**n = 534**	**n = 91**	**n = 162**	**n = 30**	**n = 123**	**n = 67**	**n = 41**	**n = 20**	**n = 288**	**n = 122**	**n = 100**	**n = 24**
**Physicians**	**377 (71)**	**40 (44)**	**138 (85)**	**9 (30)**	**120 (98)**	**17 (25)**	**40 (98)**	**13 (65)**	**183 (64)**	**73 (60)**	**98 (98)**	**23 (96)**
Physician in training (ICU)	69 (13)	4 (4)	10 (6)	0 (0)	**39 (32)**	1 (1)	10 (24)	5 (25)	14 (5)	**27 (22)**	18 (18)	**10 (42)**
Physician in training (ID)	31 (6)	2 (2)	3 (2)	0 (0)	22 (18)	0 (0)	0 (0)	4 (20)	4 (1)	11 (9)	10 (10)	6 (25)
Specialist in Intensive Care Medicine	**187 (35)**	**28 (31)**	**103 (64)**	**6 (20)**	16 (13)	**11 (16)**	**22 (54)**	1 (5)	**134 (47)**	12 (10)	**41 (41)**	0 (0)
Specialist in Infectious Diseases	30 (6)	5 (5)	14 (9)	3 (10)	3 (2)	5 (7)	0 (0)	0 (0)	22 (8)	4 (3)	4 (4)	0 (0)
Other	59 (11)	1 (1)	8 (5)	0 (0)	**39 (32)**	0 (0)	8 (20)	3 (15)	9 (3)	19 (16)	25 (25)	**7 (29)**
**Pharmacists**	**153 (29)**	**51 (56)**	**22 (14)**	**21 (70)**	**2 (2)**	**50 (75)**	**1 (2)**	**6 (30)**	**103 (36)**	**48 (39)**	**2 (2)**	**0 (0)**
ICU Pharmacist	**96 (18)**	**32 (35)**	7 (4)	**17 (57)**	1 (1)	**36 (54)**	**1 (2)**	2 (10)	**63 (22)**	**31 (25)**	**2 (2)**	0 (0)
ID Pharmacist	20 (4)	4 (4)	6 (4)	1 (3)	0 (0)	9 (13)	0 (0)	0 (0)	18 (6)	2 (2)	0 (0)	0 (0)
AMS Pharmacist	28 (5)	12 (13)	**8 (5)**	0 (0)	1 (1)	4 (6)	0 (0)	**3 (15)**	18 (6)	10 (8)	0 (0)	0 (0)
Other	9 (2)	3 (3)	1 (0.5)	3 (10)	0 (0)	1 (1)	0 (0)	1 (5)	4 (1)	5 (4)	0 (0)	0 (0)
**Others**	**4 (1)**	**0 (0)**	**2 (1)**	**0 (0)**	**1 (1)**	**0 (0)**	**0 (0)**	**1 (5)**	**2 (0.5)**	**1 (1)**	**0 (0)**	1 (4)
**Availability of a Pharmacist**	**n= 518**	**n= 91**	**n= 155**	**n= 30**	**n= 117**	**n= 66**	**n= 40**	**n= 19**	**n= 279**	**n= 121**	**n= 95**	**n= 23**
Every day	87 (17)	13 (14)	13 (8)	10 (33)	22 (19)	21 (32)	3 (8)	5 (26)	42 (15)	27 (22)	13 (14)	5 (22)
Five days per week	**188 (37)**	**70 (77)**	40 (26)	**11 (37)**	25 (21)	**41 (62)**	3 (8)	4 (21)	**128 (46)**	**53 (44)**	12 (13)	1 (4)
Phone Consultation	82 (16)	1 (1)	48 (31)	1 (3)	24 (21)	2 (3)	3 (8)	4 (21)	50 (18)	12 (10)	14 (15)	7 (30)
Other	12 (2)	0 (0)	3 (2)	1 (3)	1 (1)	2 (3)	0 (0)	0 (0)	3 (1)	1(2)	1 (1)	0 (0)
None	149 (29)	7 (8)	**51 (33)**	7 (23)	**45 (38)**	0 (0)	**31 (78)**	**6 (32)**	56 (20)	28 (23)	**55 (58)**	**10 (43)**
**ID Input**	**n = 531**	**n= 91**	**n = 161**	**n = 30**	**n = 121**	**n = 67**	**n = 41**	**n = 20**	**n = 287**	**n = 122**	**n = 98**	**n = 24**

Variable	Total	East Asia and Pacific	Europe and Central Asia	Latin America and the Caribbean	Middle East and North Africa	North America	South Asia	Sub-Saharan Africa	High-Income	Upper-Middle - Income	Lower-Middle-Income	Low-Income
At least one ICU specialist qualified in Clinical Microbiology or ID	86 (16)	3 (3)	47 (29)	6 (20)	22 (18)	3 (4)	2 (5)	3 (15)	51 (18)	13 (11)	18 (18)	4 (17)
Consultation on request only	**188 (35)**	22 (24)	40 (25)	5 (17)	**38 (31)**	**52 (78)**	**23 (56)**	**8 (40)**	99 (34)	**46 (38)**	**38 (39)**	5 (21)
Regular ID round or consultation	183 (34)	**59 (65)**	**64 (40)**	**15 (50)**	27 (22)	12 (18)	3 (7)	3 (15)	**125 (44)**	37 (30)	16 (16)	5 (21)
None	73 (14)	7 (8)	10 (6)	3 (10)	34 (28)	0 (0)	13 (32)	6 (30)	12 (4)	25 (20)	26 (27)	**10 (42)**
Other	1 (0.2)	0 (0)	0 (0)	1 (3)	0 (0)	0 (0)	0 (0)	0 (0)	0 (0)	1 (1)	0 (0)	0 (0)
**ID team members (select all)**	**n = 183**	**n = 59**	**n = 64**	**n = 15**	**n = 38**	**n = 12**	**n = 3**	**n = 3**	**n = 125**	**n = 37**	**n = 16**	**n = 5**
ID Specialist	**150 (82)**	**53 (90)**	**48 (75)**	**15 (100)**	**15 (39)**	**12 (100)**	1 (33)	**2 (67)**	**107 (86)**	**30 (82)**	9 (56)	**4 (80)**
ID Physician in Training	90 (49)	46 (78)	18 (28)	4 (27)	**15 (39)**	5 (42)	**2 (67)**	0 (0)	64 (51)	13 (35)	**10 (63)**	3 (60)
ID/AMS Pharmacist	83 (45)	38 (64)	31 (48)	4 (27)	1 (3)	8 (67)	0 (0)	1 (33)	71 (57)	12 (32)	0 (0)	0 (0)
ID/AMS Nurse	36 (20)	10 (17)	9 (14)	10 (67)	5 (13)	1 (8)	1 (33)	0 (0)	16 (13)	16 (43)	2 (13)	2 (40)
Microbiologist	79 (43)	29 (49)	42 (66)	1 (7)	3 (8)	1 (8)	**2 (67)**	1 (33)	61 (49)	13 (35)	5 (31)	0 (0)
Other	9 (5)	3 (5)	4 (6)	0 (0)	2 (5)	0 (0)	0 (0)	0 (0)	7 (6)	1 (3)	1 (6)	0 (0)
**ID Rounding frequency per week**	**n = 174**	**n = 54**	**n = 64**	**n = 15**	**n = 23**	**n = 10**	**n = 3**	**n = 2**	**n = 118**	**n = 37**	**n = 15**	**n = 4**
1 -2	**112 (64)**	**41 (76)**	**46 (72)**	**8 (53)**	8 (35)	**4 (40)**	**2 (67)**	**2 (100)**	**82 (69)**	**21 (57)**	**8 (53)**	1 (25)
3-5	51 (29)	12 (22)	15 (23)	6 (40)	**13 (57)**	2 (20)	1 (33)	0 (0)	29 (25)	14 (38)	5 (33)	**3 (75)**
> 5	11 (6)	1 (2)	3 (5)	1 (7)	2 (9)	**4 (40)**	0 (0)	0 (0)	7 (6)	2 (5)	2 (13)	0 (0)
**Access to either National or Institutional Guidelines for antibiotic dosing?**	**n = 534**	**n = 91**	**n = 162**	**n = 30**	**n = 123**	**n = 67**	**n = 41**	**n = 20**	**n = 288**	**n = 122**	**n = 100**	**n = 24**
Yes	**407 (76)**	**87 (96)**	**127 (78)**	**23 (77)**	**72 (59)**	**58 (87)**	**28 (71)**	**11 (55)**	**242 (84)**	**86 (70)**	**62 (62)**	**17 (71)**
No	127 (24)	4 (4)	35 (22)	7 (23)	51 (41)	9 (13)	12 (29)	9 (45)	46 (16)	36 (30)	38 (38)	7 (29)
**Access to Guidelines for TDM application, interpretation and dose adjustment?**	**n = 534**	**n = 91**	**n = 162**	**n = 30**	**n = 123**	**n = 67**	**n = 41**	**n = 20**	**n = 288**	**n = 123**	**n = 99**	**n = 24**

Variable	Total	East Asia and Pacific	Europe and Central Asia	Latin America and the Caribbean	Middle East and North Africa	North America	South Asia	Sub-Saharan Africa	High-Income	Upper-Middle - Income	Lower-Middle-Income	Low-Income
Yes	260 (49)	**64 (70)**	**81 (50)**	**16 (53)**	36 (29)	**54 (81)**	1 (2)	8 (40)	**171 (59)**	**62 (50)**	16 (16)	11 (46)
No	**274 (51)**	27 (30)	**81 (50)**	14 (47)	**87 (71)**	13 (19)	**40 (98)**	**12 (60)**	117 (41)	**61 (50)**	**83 (84)**	**13 (54)**
**Meropenem Infusion Duration**	**n = 384**	**n = 76**	**n = 136**	**n = 24**	**n = 47**	**n = 62**	**n = 35**	**n = 8**	**n = 244**	**n = 78**	**n = 54**	**n = 8**
Meropenem bolus	19 (5)	6 (8)	5 (4)	1 (4)	5 (11)	1 (2)	1 (3)	0 (0)	8 (3)	7 (9)	3 (6)	1 (13)
Meropenem II	145 (38)	**45 (59)**	22 (16)	9 (38)	**35 (74)**	**35 (56)**	1 (3)	2 (25)	95 (39)	30 (38)	13 (24)	**7 (88)**
Meropenem EI	**194 (51)**	25 (33)	**85 (63)**	**14 (58)**	7 (15)	26 (42)	**32 (91)**	**5 (63)**	**117 (48)**	**40 (51)**	**37 (69)**	0 (0)
Meropenem CI	26 (7)	0 (0)	24 (18)	0 (0)	0 (0)	0 (0)	1 (3)	1 (13)	24 (10)	1 (2)	1 (2)	0 (0)
**Piperacillin/tazobactam Infusion Duration**	**n = 392**	**n = 82**	**n = 143**	**n = 27**	**n = 38**	**n = 62**	**n = 32**	**n = 8**	**n = 244**	**n = 78**	**n = 54**	**n = 4**
Piperacillin/tazobactam bolus	29 (7)	9 (11)	7 (5)	1 (4)	5 (13)	5 (8)	1 (3)	1 (13)	17 (7)	9 (12)	3 (6)	0 (0)
Piperacillin/tazobactam II	142 (36)	**49 (60)**	23 (16)	9 (33)	**27 (71)**	21 (34)	12 (38)	1 (13)	85 (35)	30 (38)	**23 (43)**	**4 (100)**
Piperacillin/tazobactam EI	**166 (42)**	23 (28)	**68 (48)**	**13 (48)**	6 (16)	**34 (55)**	**19 (59)**	**3 (38)**	**108 (44)**	**35 (45)**	**23 (43)**	0 (0)
Piperacillin/tazobactam CI	55 (14)	1 (1)	45 (31)	4 (15)	0 (0)	2 (3)	0 (0)	**3 (38)**	47 (19)	5 (6)	3 (6)	0 (0)
**Vancomycin Infusion Duration**	**n = 403**	**n = 86**	**n = 137**	**n = 25**	**n = 52**	**n = 63**	**n = 31**	**n = 9**	**n = 256**	**n = 84**	**n = 57**	**n = 6**
Vancomycin II	**313 (78)**	**79 (92)**	**70 (51)**	**24 (96)**	**40 (77)**	**62 (98)**	**29 (94)**	**9 (100)**	**183 (71)**	**78 (93)**	**46 (81)**	**6 (100)**
Vancomycin CI	90 (22)	7 (8)	67 (49)	1 (4)	12 (23)	1 (2)	2 (6)	0 (0)	73 (29)	6 (7)	11 (19)	0 (0)
**Aminoglycoside Infusion Duration**	**n = 342**	**n = 68**	**n = 115**	**n = 28**	**n = 42**	**n = 53**	**n = 31**	**n = 5**	**n = 210**	**n = 71**	**n = 54**	**n = 7**
Aminoglycoside bolus	15 (4)	5 (7)	3 (3)	1 (4)	4 (10)	0 (0)	1 (3)	1 (20)	6 (3)	1 (1)	1 (2)	1 (14)
Aminoglycoside II	**314 (92)**	**60 (88)**	**109 (95)**	**24 (86)**	**36 (86)**	**53 (100)**	**28 (90)**	**4 (80)**	**200 (95)**	**47 (66)**	**47 (87)**	**6 (86)**
Aminoglycoside EI	13 (4)	3 (4)	3 (3)	3 (11)	2 (5)	0 (0)	2 (6)	0 (0)	4 (2)	6 (8)	6 (11)	0 (0)
**Vancomycin TDM utilisation**	**n = 505**	**n = 91**	**n = 161**	**n = 29**	**n = 104**	**n = 66**	**n = 37**	**n = 17**	**n = 285**	**n = 110**	**n = 88**	**n = 22**
Yes	**452 (90)**	**91 (100)**	**153 (95)**	**25 (86)**	**71 (68)**	**65 (98)**	**32 (86)**	**15 (88)**	**276 (97)**	**91 (83)**	**69 (78)**	**16 (73)**
No	53 (10)	0 (0)	8 (5)	4 (14)	33 (32)	1 (2)	5 (14)	2 (12)	9 (3)	19 (17)	19 (22)	6 (27)
**Vancomycin dosing software utilisation**	**n = 535**	**n = 91**	**n = 162**	**n = 30**	**n = 124**	**n = 67**	**n = 41**	**n = 20**	**n = 288**	**n = 124**	**n = 99**	**n = 24**

Variable	Total	East Asia and Pacific	Europe and Central Asia	Latin America and the Caribbean	Middle East and North Africa	North America	South Asia	Sub-Saharan Africa	High-Income	Upper-Middle - Income	Lower-Middle-Income	Low-Income
**Yes**	**60 (11)**	15 (16)	22 (14)	2 (7)	4 (3)	**14 (21)**	0 (0)	3 (15)	50 (17)	5 (4)	2 (2)	3 (13)
**No**	475 (89)	76 (84)	140 (86)	28 (93)	120 (97)	53 (79)	41 (100)	17 (85)	238 (83)	119 (96)	97 (98)	21 (88)
**Piperacillin/tazobactam TDM utilisation**	**n = 460**	**n = 75**	**n = 145**	**n = 29**	**n = 105**	**n = 56**	**n = 36**	**n = 14**	**n = 250**	**n = 101**	**n = 89**	**n = 20**
Yes	199 (43)	14 (19)	**87 (60)**	**16 (55)**	50 (48)	4 (7)	**18 (50)**	**10 (71)**	99 (40)	40 (40)	**47 (53)**	**13 (65)**
No	**261 (57)**	**61 (81)**	58 (40)	13 (45)	**55 (52)**	**52 (93)**	**18 (50)**	4 (29)	**151 (60)**	**61 (60)**	42 (47)	7 (35)
**Meropenem TDM utilisation**	**n = 454**	**n = 77**	**n = 142**	**n = 27**	**n = 101**	**n = 58**	**n = 33**	**n = 16**	**n = 251**	**n = 102**	**n = 82**	**n = 19**
Yes	179 (39)	14 (18)	**83 (58)**	**14 (52)**	38 (38)	4 (7)	**18 (55)**	**8 (50)**	97 (39)	33 (32)	40 (49)	9 (47)
No	**275 (61)**	**63 (82)**	59 (42)	13 (48)	**63 (62)**	**54 (93)**	15 (45)	**8 (50)**	**154 (61)**	**69 (68)**	**42 (51)**	**10 (53)**
**Beta lactam dosing software utilisation**	**n = 506**	**n = 86**	**n = 152**	**n = 30**	**n =118**	**n = 61**	**n = 40**	**n = 19**	**n = 268**	**n = 118**	**n = 97**	**n = 23**
**Yes**	**15 (3)**	**1 (1)**	7 (5)	1 (3)	**6 (5)**	**0 (0)**	0 (0)	**0 (0)**	**8 (3)**	**1 (1)**	**5 (5)**	**1 (4)**
**No**	**491 (97)**	**85 (99)**	145 (95)	29 (97)	**112 (95)**	**61 (100)**	40 (100)	**19 (100)**	**260 (97)**	**117 (99)**	**92 (95)**	**22 (96)**
**Aminoglycoside TDM utilisation**	**n = 471**	**n =87**	**n = 147**	**n = 22**	**n = 102**	**n = 66**	**n = 33**	**n = 14**	**n = 270**	**n = 99**	**n = 81**	**n = 21**
**Yes**	**386 (82)**	**78 (90)**	**134 (91)**	**18 (82)**	**62 (61)**	**61 (92)**	**21 (64)**	**12 (86)**	**244 (90)**	**70 (71)**	**57 (70)**	**15 (71)**
**No**	85 (18)	9 (10)	13 (9)	4 (18)	40 (39)	5 (8)	12 (36)	2 (14)	26 (10)	29 (29)	24 (30)	6 (29)
**Aminoglycoside dosing software utilisation**	**n = 499**	**n = 89**	**n = 151**	**n = 25**	**n = 113**	**n = 64**	**n = 37**	**n = 17**	**n = 273**	**n = 113**	**n = 89**	**n = 21**
**Yes**	38 (8)	16 (18)	14 (9)	1 (1)	3 (3)	3 (5)	0 (0)	1 (6)	33 (12)	2 (2)	2 (2)	1 (5)
**No**	461 (92)	73 (82)	137 (91)	24 (99)	110 (97)	61 (95)	37 (100)	16 (94)	240 (88)	111 (98)	87 (98)	20 (95)
Vancomycin LD administered?	**n = 403**	**n = 86**	**n = 137**	**n = 25**	**n = 52**	**n = 63**	**n = 31**	**n = 9**	**n = 256**	**n =84**	**n = 57**	**n = 6**
Yes	**300 (74)**	**74 (86)**	**106 (77)**	**20 (80)**	25 (48)	**51 (81)**	**18 (58)**	**6 (67)**	**206 (80)**	**58 (69)**	**35 (61)**	1 (17)
No	103 (26)	12 (14)	31 (23)	5 (20)	**27 (52)**	12 (19)	13 (42)	3 (33)	50 (20)	26 (31)	22 (39)	**5 (83)**
**Vancomycin LD (II)**	**n = 220**	**n = 67**	**n = 46**	**n = 18**	**n = 18**	**n = 50**	**n = 15**	**n = 6**	**n = 140**	**n = 54**	**n = 25**	**n = 1**
mg/kg median (IQR)	25 (19-25)	25 (25-30)	20 (18-25)	25 (20-25)	15 (13-24)	25 (23-25)	25 (20-26)	25 (16-29)	25 (20-25)	25 (18-25)	20 (15-25)	12.5
**Vancomycin LD (CI)**	**n = 80**	**n = 7**	**n = 60**	**n = 2**	**n = 7**	**n = 1**	**n = 3**	**n = 0**	**n = 66**	**n = 4**	**n = 10**	**n = 0**

Variable	Total	East Asia and Pacific	Europe and Central Asia	Latin America and the Caribbean	Middle East and North Africa	North America	South Asia	Sub-Saharan Africa	High-Income	Upper-Middle -Income	Lower-Middle-Income	Low-Income
mg/kg median (IQR)	20 (13-25)	25 (23-28)	20 (13-25)	16.5 (14-18)	25 (15-28)	25	25 (19-25)	N/A	20 (13-25)	17.5 (15-21)	25 (16-25)	N/A
**Vancomycin Obese LD capped?**	**n = 508**	**n = 91**	**n = 158**	**n = 29**	**n = 108**	**n = 66**	**n = 38**	**n = 18**	**n = 281**	**n = 113**	**n = 92**	**n = 22**
Yes	**366 (72)**	**86 (95)**	**114 (72)**	**23 (79)**	43 (40)	**65 (98)**	**24 (63)**	**11 (61)**	**233 (83)**	**81 (72)**	42 (46)	10 (45)
No	142 (28)	5 (5)	44 (28)	6 (21)	**65 (60)**	1 (2)	14 (37)	7 (39)	48 (17)	32 (28)	**50 (54)**	**12 (55)**
**Vancomycin Obese LD**	**n = 420**	**n = 89**	**n = 138**	**n = 29**	**n = 61**	**n = 65**	**n = 32**	**n = 6**	**n = 262**	**n = 85**	**n = 66**	**n = 7**
mg/kg median (IQR)	12.5 (10-15)	15 (10-15)	10 (10-15)	15 (10-20)	10 (8-15)	12.5 (10-15)	12.5 (10-15)	10 (10-14)	12.5 (10-15)	10 (10-15)	15 (10-20)	15 (9-18)
**Vancomycin MD (II)**	**n = 313**	**n = 79**	**n = 70**	**n = 24**	**n = 40**	**n = 62**	**n = 29**	**n = 9**	**n = 183**	**n = 78**	**n = 46**	**n = 6**
mg/kg/24 hr (median (IQR))	35 (25-40)	35 (29-38)	31 (25-38)	27.5 (25-41)	30 (25-44)	40 (31-46)	30 (25-40)	37.5 (25-50)	37.5 (30-40)	30 (25-38)	30 (25-40)	27.5 (16-38)
**Vancomycin MD (CI)**	**n = 90**	**n = 7**	**n = 67**	**n = 1**	**n = 12**	**n = 1**	**n = 2**	**n = 0**	**n = 73**	**n = 6**	**n =11**	**n = 0**
mg/kg/24 hr (median (IQR))	30 (25-31)	30 (28-30)	30 (25-31)	15	30 (24-30)	31	22.5 (19-26)	N/A	30 (25-31)	30 (22-30)	30 (23-30)	N/A
**Amikacin* dose mg/kg ABW**	**n = 100**	**n = 13**	**n = 34**	**n = 11**	**n = 11**	**n = 5**	**n = 25**	**n = 5**	**n = 41**	**n = 27**	**n = 35**	**n = 1**
Amikacin* dose mg/kg ABW (median(IQR))	15 (13-20)	15 (13-15)	20 (15-25)	12.5 (13-15)	15 (11-17)	20 (19-20)	12.5 (9-15)	15 (13-18)	20 (15-25)	15 (13-15)	12.5 (11-15)	5
**Gentamicin* dose mg/kg ABW**	**n = 104**	**n = 33**	**n = 46**	**n = 4**	**n = 9**	**n = 11**	**n = 1**	**n = 0**	**n = 85**	**n = 10**	**n = 8**	**n = 1**
**Gentamicin* dose mg/kg ABW (median(IQR))**	5 (4-7)	6 (5-7)	5 (4-6)	4.5 (4-5)	4 (3-7)	7 (5-7)	4	N/A	6 (5-7)	4 (3-5)	4.5 (4-7)	3
**Tobramycin* dose mg/kg ABW**	**n = 26**	**n = 0**	**n = 5**	**n = 0**	**n = 0**	**n = 21**	**n = 0**	**n = 0**	**n = 26**	**n = 0**	**n = 0**	**n = 0**
Tobramycin* dose mg/kg ABW (median (IQR))	5.6 (5-7)	N/A	5 (3-5)	N/A	N/A	7 (5-7)	N/A	N/A	5.6 (5-7)	N/A	N/A	N/A
**Vancomycin PK/PD target (CI)**	**n = 69**	**n = 4**	**n = 55**	**n = 0**	**n = 7**	**n = 1**	**n = 2**	**n = 0**	**n = 59**	**n = 3**	**n = 7**	**n = 0**
AUC/MIC target 400 -600	9 (13)	0 (0)	9 (16)	0 (0)	0 (0)	0 (0)	0 (0)	0 (0)	9 (15)	0 (0)	0 (0)	0 (0)
**Css target**	**60 (87)**	**4 (100)**	**46 (84)**	**0 (0)**	**7 (100)**	**1 (100)**	**2 (100)**	**0 (0)**	**50 (85)**	**3 (100)**	**7 (100)**	**0 (0)**
Css 10-15 mg/L	2 (3)	0 (0)	0 (0)	0 (0)	1 (14)	0 (0)	1 (50)	0 (0)	0 (0)	1 (33)	2 (29)	0 (0)

Variable	Total	East Asia and Pacific	Europe and Central Asia	Latin America and the Caribbean	Middle East and North Africa	North America	South Asia	Sub-Saharan Africa	High-Income	Upper-Middle - Income	Lower-Middle-Income	Low-Income
Css 15-20 mg/L	11 (16)	2 (50)	7 (13)	0 (0)	1 (14)	0 (0)	**1 (50)**	0 (0)	8 (14)	**2 (67)**	2 (29)	0 (0)
**Css 20-25 mg/L**	**45 (65)**	**2 (50)**	**37 (67)**	**0 (0)**	**5 (71)**	**1 (100)**	0 (0)	**0 (0)**	**40 (68)**	0 (0)	**3 (43)**	**0 (0)**
Other	2 (3)	0 (0)	2 (4)	0 (0)	0 (0)	0 (0)	0 (0)	0 (0)	2 (3)	0 (0)	0 (0)	0 (0)
**Vancomycin PK/PD target (II)**	**n = 380**	**n = 87**	**n = 95**	**n = 24**	**n = 64**	**n = 65**	**n = 30**	**n = 15**	**n = 215**	**n = 87**	**n = 61**	**n = 17**
AUC/MIC target 400 -600	80 (21)	21 (24)	12 (13)	5 (21)	12 (19)	22 (34)	3 (10)	5 (33)	42 (20)	25 (29)	7 (11)	**6 (35)**
**Trough target**	**300 (79)**	**66 (76)**	**83 (87)**	**19 (79)**	**52 (81)**	**43 (66)**	**27 (90)**	**10 (67)**	**173 (80)**	**62 (71)**	**54 (89)**	**11 (65)**
**Trough 10-15 mg/L**	36 (9)	4 (5)	5 (5)	2 (10)	9 (14)	11 (17)	5 (17)	0 (0)	17 (8)	8 (9)	11 (18)	0 (0)
Trough 12-18mg/L	31 (8)	2 (2)	8 (8)	0 (0)	11 (17)	6 (9)	2 (7)	2 (13)	16 (7)	6 (7)	7 (11)	2 (12)
Trough 15-20 mg/L	**183 (48)**	**54 (62)**	**49 (52)**	**16 (67)**	**18 (28)**	**22 (34)**	**18 (60)**	**6 (40)**	**112 (52)**	**42 (48)**	**27 (44)**	2 (12)
Trough 20-25 mg/L	40 (11)	3 (3)	19	1 (4)	14 (22)	0 (0)	2 (7)	1 (7)	20 (9)	5 (6)	9 (15)	**6 (35)**
Other	10 (3)	3 (3)	2	0 (0)	0 (0)	4 (6)	0 (0)	1 (7)	8 (4)	1 (1)	0 (0)	1 (6)
**Piperacillin/tazobactam PK/PD target**	**n = 258**	**n = 40**	**n = 96**	**n = 13**	**n = 47**	**n = 32**	**n = 18**	**n = 12**	**n = 154**	**n = 47**	**n = 45**	**n = 12**
100% fT>4xMIC	59 (23)	3 (8)	**42 (44)**	2 (15)	4 (9)	3 (9)	1 (6)	4 (33)	48 (31)	5 (11)	3 (7)	3 (25)
100% fT>MIC	**92 (36)**	**21 (53)**	27 (28)	**6 (46)**	11 (23)	**19 (59)**	3 (17)	**5 (42)**	**60 (39)**	**19 (40)**	9 (20)	**4 (33)**
50% fT>4xMIC	46 (18)	10 (25)	15 (16)	2 (15)	7 (15)	5 (16)	**6 (33)**	1 (8)	27 (18)	3 (6)	**15 (33)**	1 (8)
50% fT>MIC	33 (13)	2 (5)	6 (6)	1 (8)	**14 (30)**	3 (9)	**6 (33)**	1 (8)	8 (5)	10 (21)	14 (31)	1 (8)
AUC/MIC	11 (4)	0 (0)	1 (1)	0 (0)	8 (17)	0 (0)	2 (11)	0 (0)	1 (0.5)	5 (11)	3 (7)	2 (17)
CMax/MIC	1 (0.5)	0 (0)	0 (0)	0 (0)	1 (2)	0 (0)	0 (0)	0 (0)	0 (0)	1 (2)	0 (0)	0 (0)
Unsure	13 (5)	4 (10)	2 (2)	2 (15)	2 (4)	2 (6)	0 (0)	1 (8)	7 (5)	4 (9)	1 (2)	1 (8)
Other	3 (1)	0 (0)	3 (3)	0 (0)	0 (0)	0 (0)	0 (0)	0 (0)	3 (2)	0 (0)	0 (0)	0 (0)
**Meropenem PK/PD target**	**n = 204**	**n = 24**	**n = 86**	**n = 12**	**n = 38**	**n = 8**	**n = 17**	**n = 7**	**n = 109**	**n = 38**	**n = 37**	**n = 8**
100% fT>4xMIC	52 (25)	4 (17)	**36 (42)**	1 (8)	7 (18)	**3 (38)**	0 (0)	1 (14)	**43 (39)**	2 (5)	4 (11)	**3 (38)**
100% fT>MIC	**57 (28)**	**12 (50)**	25 (29)	**3 (25)**	6 (16)	**3 (38)**	**6 (35)**	**2 (29)**	36 (33)	**11 (29)**	**10 (27)**	0 (0)
50% fT>4xMIC	27 (13)	3 (13)	13 (15)	1 (8)	5 (13)	0 (0)	4 (24)	1 (7)	13 (12)	5 (13)	8 (22)	1 (13)
50% fT>MIC	29 (14)	1 (4)	7 (8)	**3 (25)**	**10 (26)**	1 (13)	5 (29)	**2 (29)**	9 (8)	8 (21)	**10 (27)**	2 (25)

Variable	Total	East Asia and Pacific	Europe and Central Asia	Latin America and the Caribbean	Middle East and North Africa	North America	South Asia	Sub-Saharan Africa	High-Income	Upper-Middle - Income	Lower-Middle-Income	Low-Income
AUC/MIC	9 (4)	0 (0)	1 (1)	1 (8)	4 (11)	0 (0)	2 (12)	1 (7)	1 (1)	4 (11)	2 (5)	2 (25)
CMax/MIC	5 (2)	0 (0)	0 (0)	1 (8)	4 (11)	0 (0)	0 (0)	0 (0)	0 (0)	4 (11)	1 (3)	0 (0)
Unsure	11 (5)	4 (17)	2 (2)	2 (17)	2 (5)	1 (13)	0 (0)	0 (0)	5 (5)	4 (11)	2 (5)	0 (0)
Other	2 (1)	0 (0)	2 (2)	0 (0)	0 (0)	0 (0)	0 (0)	0 (0)	2 (2)	0 (0)	0 (0)	0 (0)
**Aminoglycoside PK/PD target**	**n = 385**	**n = 77**	**n = 132**	**n = 22**	**n = 63**	**n = 58**	**n = 21**	**n = 12**	**n = 238**	**n = 76**	**n = 56**	**n = 15**
100% fT>MIC	24 (6)	2 (26)	8 (6)	3 (14)	4 (6)	3 (5)	2 (10)	2 (17)	11 (5)	6 (8)	3 (5)	4 (27)
AUC/MIC	65 (17)	15 (19)	15 (11)	1 (5)	17 (27)	10 (17)	5 (24)	2 (17)	33 (14)	12 (16)	16 (29)	4 (27)
CMax/MIC	**166 (43)**	**35 (45)**	**67 (51)**	**9 (41)**	7 (11)	34 (59)	**12 (57)**	2 (17)	**119 (50)**	**29 (38)**	18 (32)	0 (0)
Unsure	130 (34)	25 (32)	42 (32)	**9 (41)**	**35 (56)**	11 (19)	2 (10)	**6 (50)**	75 (32)	**29 (38)**	**19 (34)**	**7 (47)**

Abbreviations: ICU, intensive care unit; ID, infectious diseases; AMS, antimicrobial stewardship; TDM, therapeutic drug monitoring; II, intermittent infusion; EI, extended infusion; CI, continuous infusion; LD, loading dose; *IQR, interquartile range; MD, maintenance dose; *, once daily dosing; ABW, actual body weight;* AUC, area under the curve; MIC, minimum inhibitory *concentration; fT, free time; Css, concentration at steady state; PK, pharmacokinetics; PD, pharmacodynamics; CMax, concentration maximum; n, number; %, percentage.*

Table S6: TDM utilization and beta-lactam prolonged infusion administration according to hospital type

Antibiotic	Academic Hospital*	General Hospital	P value
	TDM utilisation		
Vancomycin	92% (275/300)	86% (153/175)	**0.049**
Piperacillin/Tazobactam	50% (137/276)	34% (54/160)	**0.001**
Meropenem	44% (118/268)	32% (52/162)	**0.014**
Aminoglycosides	84% (237/284)	79% (128/162)	0.242
	Prolonged infusion$^{\alpha}$ utilisation		
Piperacillin/Tazobactam	54% (132/243)	52% (75/145)	0.620
Meropenem	55% (132/239)	50% (74/149)	0.285

** academic hospital includes university and university affiliated hospital; α prolonged infusion includes extended infusion and continuous infusion*
Abbreviations: TDM, therapeutic drug monitoring

Table S7: Comparable results (ADMIN ICU 2021 and ADMIN ICU 2015)

**Variable**	**Adjusted variable used in ADMIN-ICU 2015**	**ADMIN-ICU 2021**	**ADMIN-ICU 2015**
		n (%) or median (IQR)	n (%) or median (IQR)
Respondents		538	402
Hospitals		409	328
Cities		292	252
Countries		45	53
**Experience in ICU**		**n = 533**	**n = 402**
< 5 years		258 (48)	74 (18)
5-10 years	5-15 years	131 (25)	186 (46)
> 10 years	>15 years	144 (27)	142 (35)
**Type of hospital**		**n = 529**	**n = 402**
General		188 (36)	132 (33)
Rural		13 (2)	0 (0)
University		199 (38)	150 (37)
University affiliated		123 (23)	120 (30)
Other		6 (1)	0 (0)
**ICU type**		**n = 528**	**n = 402**
Cardiac		9 (2)	8 (2)
Medical		99 (19)	37 (9)
Medical-Surgical		342 (65)	318 (79)
Surgical		48 (9)	22 (6)
Other		30 (6)	17 (4)
**Open of Closed ICU**		**n = 532**	**n = 402**
Closed		339 (64)	269 (67)
Open		193 (37)	133 (33)
**Position**		**n = 534**	**n = 402**
**Physicians**		**377 (71)**	**342 (85)**
Physician in training		100 (19)	28 (7)

Variable	Adjusted variable used in ADMIN-ICU 2015	ADMIN-ICU 2021	ADMIN-ICU 2015
Specialist in Intensive Care Medicine or Infectious Diseases		**217 (35)**	**314 (78)**
Pharmacists		153 (29)	48 (12)
Others		4 (1)	12 (3)
**Availability of a Pharmacist**		**n= 518**	**n = 402**
Every day		87 (17)	169 (42)
Five days per week		**188 (37)**	**N/A**
Phone Consultation		82 (16)	83 (21)
Other		12 (2)	39 (10)
None		149 (29)	111 (28)
**Access to either National or Institutional Guidelines for antibiotic dosing?**		**n = 534**	**n = 401**
Yes		**407 (76)**	**235 (59)**
No		127 (24)	166 (41)
**Access to Guidelines for TDM application, interpretation and dose adjustment?**		**n = 534**	**N/A**
Yes		260 (49)	N/A
No		**274 (51)**	**N/A**
**Meropenem Infusion Duration**		**n = 384**	**n = 396**
Meropenem bolus		19 (5)	N/A
Meropenem II		145 (38)	**268 (68)**
Meropenem EI		**194 (51)**	111 (28)
Meropenem CI		26 (7)	17 (4)
**Piperacillin/tazobactam Infusion Duration**		**n = 392**	**n = 402**
Piperacillin/tazobactam bolus		29 (7)	N/A
Piperacillin/tazobactam II		142 (36)	291 (72)
Piperacillin/tazobactam EI		**166 (42)**	**83 (21)**
Piperacillin/tazobactam CI		55 (14)	28 (7)
**Vancomycin Infusion Duration**		**n = 403**	**n = 402**
Vancomycin II		**313 (78)**	**276 (69)**
Vancomycin CI		90 (22)	126 (31)

Variable	Adjusted variable used in ADMIN-ICU 2015	ADMIN-ICU 2021	ADMIN-ICU 2015
**Aminoglycoside once daily infusion Duration**		**n = 342**	**N/A**
Aminoglycoside bolus		15 (4)	N/A
Aminoglycoside II	Median (IQR) infusion duration (min)	**314 (92)**	60 (30-60) amikacin<br>30 (30-60) gentamicin
Aminoglycoside EI		13 (4)	N/A
**Preferred aminoglycoside**		n = 317	n = 402
Gentamicin		149 (47)	221 (55)
Amikacin		128 (40)	181 (45)
Tobramycin		40 (13)	20 (5)
**Aminoglycoside dosing frequency**		**n = 390**	**n = 402**
Once daily		331 (85)	378 (94)
Divided daily dose		59 (15)	24 (6)
**Vancomycin LD administered?**		**300/403 (74)**	**261/402 (65)**
**Vancomycin LD (II)**		**n = 220**	**n = 274**
mg/kg median (IQR)		25 (19-25)	23 (19-25)
**Vancomycin LD (CI)**		**n = 80**	**n = 120**
mg/kg median (IQR)		20 (13-25)	15 (13–20)
**Vancomycin MD (II)**		**n = 313**	**n = 267**
mg/kg/24 hr (median (IQR))		35 (25-40)	25 (15-25)
**Vancomycin MD (CI)**		**n = 90**	**n = 115**
mg/kg/24 hr (median (IQR))		30 (25-31)	25 (25-30)
**Meropenem LD administered (II)**		38/145 (26)	13/268 (5)
**Meropenem LD administered (EI)**		120/194 (62)	42/111 (38)
**Meropenem LD administered (CI)**		24/26 (92)	12/17 (73)
**Meropenem maintenance dose per 24 hours (median (IQR))**			
Meropenem bolus (g)		3 (3-6) (n = 19)	N/A
Meropenem II (g)		3 (3-4) (n = 145)	3 (3-3) (n = 261)
Meropenem EI (g)		3.5 (3-6) (n = 194)	3 (3-6) (n = 110)
Meropenem CI (g)		4 (3-6) (n = 26)	4 (3-5) (n = 17)

Variable	Adjusted variable used in ADMIN-ICU 2015	ADMIN-ICU 2021	ADMIN-ICU 2015
**Piperacillin/tazobactam LD administered (II)**		37/142 (26)	N/A
**Piperacillin/tazobactam LD administered (EI)**		103/166 (62)	27/83 (33)
**Piperacillin/tazobactam LD administered (CI)**		46/55 (84)	23/28 (82)
**Piperacillin/tazobactam dose per 24 hours (median (IQR))**			
Piperacillin/tazobactam bolus (g)		18 (13.5 – 18) (n = 29)	N/A
Piperacillin/tazobactam II (g)		18 (13.5 – 18) (n = 142)	18 (14.6 -18) (n = 288)
Piperacillin/tazobactam EI (g)		18 (13.5 – 18) (n = 166)	18 (13.5 -18) (n = 81)
Piperacillin/tazobactam CI (g)		18 (13.5 – 18) (n = 55)	18 (15.8 – 18) (n = 28)
**Amikacin* dose mg/kg ABW**	Weight metric not specified	**n = 100**	**n = 151**
Amikacin* dose mg/kg ABW (median (IQR))		15 (13-20)	15 (15-20)
**Gentamicin* dose mg/kg ABW**	Weight metric not specified	**n = 104**	**n = 208**
Gentamicin* dose mg/kg ABW (median (IQR))		5 (4-7)	5 (5-6)
**Tobramycin* dose mg/kg ABW**	Weight metric not specified	**n = 26**	**n = 19**
Tobramycin* dose mg/kg ABW (median (IQR))		5.6 (5-7)	6 (5-7)
**Vancomycin TDM utilisation**		**n = 505**	**n = 402**
Yes		**452 (90)**	**330 (82)**
No	Infrequent or never	53 (10)	72 (18)
**Piperacillin/tazobactam TDM utilisation**		**n = 460**	**n = 402**
Yes		199 (43)	40 (10)
No	Infrequent or never	**261 (57)**	**362 (90)**
**Meropenem TDM utilisation**	**Carbapenem TDM utilisation**	**n = 454**	**n = 402**
Yes		179 (39)	32 (8)
No	Infrequent or never	**275 (61)**	**370 (92)**
**Aminoglycoside TDM utilisation**		**n = 471**	**n = 402**
**Yes**		**386 (82)**	**322 (80)**
**No**	Infrequent or never	85 (18)	80 (20)
**Aminoglycoside TDM peak and trough concentration targets**			
Amikacin peak (mg/L)		38 (23-55) (n = 72)	41 (26-60) (n = 47)
Amikacin trough (mg/L)		3 (2-5) (n = 74)	3 (2-5) (n = 59)
Gentamicin peak (mg/L)		10 (8-19) (n = 69)	12 (10-17.5) (n = 37)
Gentamicin trough (mg/L)		1 (1-2) (n = 103)	1 (0.5-1.5) (n = 122)

Variable	Adjusted variable used in ADMIN-ICU 2015	ADMIN-ICU 2021	ADMIN-ICU 2015
Tobramycin peak (mg/L)		10 (9-16) (n = 25)	12 (10-23.8) (n = 7)
Tobramycin trough (mg/L)		1 (1-2) (n = 32)	2 (1.3-2) (n = 12)
**If the trough concentration is above your target, do you: (select all that apply)**		**n = 362**	**n = 193**
Leave the daily dose unchanged		44 (12)	8 (4)
Decrease the next daily dose		137 (38)	81 (42)
Delay next dose until the trough is below the target concentration		78 (22)	106 (55)
Extend the dosing frequency		120 (33)	N/A
Use dosing software to guide therapy		43 (12)	N/A

Abbreviations: ICU, intensive care unit; TDM, therapeutic drug monitoring; II, intermittent infusion; EI, extended infusion; CI, continuous infusion; LD, loading dose; *IQR, interquartile range; MD, maintenance dose; *, once daily dosing; ABW, actual body weight; n, number; %, percentage*
